# Patient–Provider Health Communication Strategies: Enhancing HPV Vaccine Uptake among Adolescents of Color

**DOI:** 10.3390/healthcare11121702

**Published:** 2023-06-10

**Authors:** Mia Ann Xu, Jasmin Choi, Ariadna Capasso, Ralph DiClemente

**Affiliations:** 1 Department of Social and Behavioral Sciences, School of Global Public Health, New York University, New York, NY 10003, USA; 2 Health Resources in Action, Boston, MA 02116, USA

**Keywords:** health communication, vaccine communication, HPV vaccine uptake, marginalized racial and ethnic groups, adolescent health, culturally adapted messaging, healthcare providers

## Abstract

Cervical cancer remains a public health issue in the United States, particularly among stigmatized racial and ethnic populations. The human papillomavirus (HPV) vaccine has been clinically proven to prevent cervical cancers, and other HPV-associated cancers, among men and women. However, HPV vaccine uptake is suboptimal; only 55% of adolescents complete the two-dose series by age 15. Past research has shown that provider HPV vaccine communication for people of marginalized races/ethnicities is subpar. This article focuses on provider communication strategies to promote HPV vaccine uptake effectively and equitably. The authors reviewed the literature on evidence-based patient–provider HPV vaccine communication techniques to create a set of communication language providers could use and avoid using to enhance HPV vaccine acceptance and uptake among adolescents of marginalized racial and ethnic groups. Evidence has shown that information and the manner of dissemination are critical for influencing HPV vaccine uptake. These communication strategies must be suited to the context of the targeted population, and the message content can be broadly categorized into source, content, and modality. Strategies to improve patient–provider communication among adolescents of color using source, modality, and content include the following: (1) Source: increase provider self-efficacy to provide the recommendation, building rapport between providers and parents; (2) Content: persistent, forceful language with minimal acquiescence should be employed, reframing the conversation focus from sex to cancer; and (3) Modality: use multiple vaccine reminder modalities, and work with the community to culturally adapt the vaccination language. Utilizing effective behavior-change communication adapted for adolescents of color can reduce missed opportunities for HPV prevention, potentially decreasing racial and ethnic disparities in HPV-related morbidity and mortality.

## 1. Introduction

The HPV vaccine has been clinically proven to prevent cervical cancer, the fourth most common cancer among women worldwide [1], as well as other HPV-associated cancers, including oropharyngeal and anogenital cancer [2]. The HPV vaccine can prevent over 90% of cancers and pre-cancers caused by HPV among both males and females [3] and has low adverse risk [4,5]. To prevent HPV, the Advisory Committee on Immunization Practices (ACIP) recommends that adolescents receive a two-dose HPV vaccine series, starting at age 11 or 12 years [3,6]. In 2019, among U.S. adolescents aged 13 to 17 years, 56.8% of females and 51.8% of males were up-to-date on the HPV vaccination [7]. To achieve the Healthy People 2030 goal of having 80% of adolescents vaccinated for HPV, action is urgently needed to accelerate HPV vaccine uptake [8]. This missed opportunity is concerning, as the HPV vaccine has an equally low adverse reaction rate as other recommended vaccines with higher vaccination rates [9,10].

In the United States, marginalized racial and ethnic populations experience significantly greater morbidity and mortality outcomes related to HPV. This is attributable to early sexual initiation, disparate rates of HPV vaccination, and the lack of timely detection and treatment of cancers [1,11]. Race and ethnicity status are known predictors of late-stage cervical cancer diagnosis and mortality [12]. While studies have found that HPV vaccination initiation rates are high among Latinx, Black, Asian, and American Indian/Alaskan Native adolescents compared to white adolescents [8,13], these marginalized racial and ethnic groups may have lower completion rates than the national average [13,14,15,16]. This may be related to issues, such as low parental knowledge about vaccine dosing or scheduling, lack of clinician reminders, and inadequate patient–provider communication [14]. 

Healthcare providers (HCPs) play an important role in educating parents about vaccine timing and vaccination completion. Regardless of racial and ethnic group, studies have found that HCP recommendation is one of the strongest and most consistent predictors of HPV vaccination initiation and completion [16,17,18,19,20], potentially even more than race or socioeconomic status [14]. However, minoritized populations report lower rates of HPV vaccine recommendation from HCPs compared to whites [14,21,22] and also often report receiving no information about subsequent necessary doses compared to whites [17]. Furthermore, research shows that HCP recommendation mediated the association between HPV awareness among parents and HPV vaccine initiation and completion in their children [23]. As the key target group for the HPV vaccine is currently 11- to 12-year-olds, parents are key decision-makers in the conversation. Many individuals state that HCPs are their primary source of information about vaccines, and talking to an HCP is their preferred way of gaining information on the HPV vaccine [24,25,26]. As awareness of the HPV vaccine is not enough to motivate actual vaccination rates among minoritized identities [27], provider recommendations may be crucial to increasing the chances of action [23]. Consequently, HCPs need to recognize that their HPV vaccine recommendation has a pivotal role in determining adolescents’ vaccine outcome and prioritize providing HPV vaccine recommendation communications that ensure equitable vaccine uptake.

One key strategy to improve patient–provider communication for behavioral-change communication is to formulate the communication messaging so that the message’s source, content, and modality are suited to the context of the targeted population, a method used in prior health works [28,29,30]. Research shows that HCPs who have completed a HPV vaccine communication training reported that the communication strategies were easy to use, saved time, and enabled them to increase their adolescent patients’ HPV vaccine uptake [31]. Tailoring health communication to context and culture is vital to increasing the message’s receptivity. This commentary describes best practices with the critical communication elements of source, content, and modality to highlight effective evidence-based communication strategies that promote equitable, culturally adapted HPV vaccine uptake among minoritized identities. 

## 2. Materials and Methods

The authors conducted a review of research-backed guidelines for effective health communications oriented toward marginalized racial and ethnic groups. The keywords for conducting the literature review included HPV vaccine messages to enhance HCPs’ development of culturally and racially adapted messaging for adolescents in the United States. The authors reviewed the 75 most relevant peer-reviewed scientific literature on effective evidence-based strategies to enhance HPV vaccine health communications. All articles were collected on PubMed from 2010 to present. The articles included adolescents, defined as those under 18 years of age. The article’s health communication strategies and references are grounded in research adapted to Latinx, Black, Asian, and American Indian/Alaskan Native groups. The review includes peer-reviewed qualitative and quantitative research that specifies the source of the messaging, the content of the messaging, and the modality used to enhance the messaging. 

## 3. Results

### 3.1. Source

#### 3.1.1. Increase Provider’s Self-Efficacy to Provide Vaccine Recommendation

As discussed, HCP recommendation is vital to increasing adolescent HPV vaccine uptake. However, some HCPs may not provide it consistently, particularly to patients of stigmatized racial and ethnic groups. The literature highlights two major reasons that make HCPs less likely to recommend the HPV vaccine. (1) HCPs who perceive that they do not identify with or have inadequate knowledge of the culture and/or religion of their patient may be less comfortable discussing vaccination with the patient. (2) HCPs have low confidence in their recommendation and ability to discuss the HPV vaccine. When considering how to discuss HPV vaccines with those from stigmatized racial and ethnic groups, providers tended to focus on the anticipated difficulties and frustrations associated with such discussions [32,33,34]. HCPs perceived that challenges in cross-cultural communication due to lack of familiarity with the family’s religious and ethnic background would negatively impact their HPV communication and recommendation [35,36]. HCPs may also not provide a strong vaccine recommendation due to a lack of confidence in their knowledge or capability to discuss HPV with the adolescent and their parents [17]. A knowledge assessment of HCPs found that over 40% did not know important HPV vaccine details, including the dosing intervals and schedules [37].

The lack of knowledge and confidence in discussing the safety and recommendation guidelines about the HPV vaccine, coupled with the lack of cultural sensitivity, is particularly detrimental to families from stigmatized racial and ethnic groups. A history of institutional racism and discrimination has led to mistrust of the healthcare system by marginalized racial and ethnic populations [38,39]. Historically marginalized racial and ethnic groups often desire more information about HPV and the vaccine prior to making their vaccination decision [40,41]. While studies have found that Latinx, Black, and Asian adults may have generally heard about HPV, these groups often have significantly lower specific knowledge about HPV, such as the number of doses required for vaccination completion and the potential severity of HPV infection, compared to white adults [17]. Parents of stigmatized racial and ethnic populations also have concerns about the vaccine’s side effects and would prefer to discuss their concerns with their HCP [40]. Unfortunately, some parents in stigmatized racial and ethnic groups perceive that their HCPs are unwilling to provide complete information on the vaccine and to answer their questions in detail, discouraging them from vaccinating their children [41].

To provide equitable HPV vaccine uptake, HCPs need to develop greater knowledge and self-efficacy in discussing HPV vaccines and to do so in culturally and religiously sensitive ways. HCP-targeted training sessions may improve provider capacity to make vaccine recommendations. Educational workshops focused on developing provider HPV and HPV vaccine knowledge, cultural competency, communication techniques, and recommendation style have been shown to help improve provider HPV knowledge, self-efficacy to recommend, intention to recommend, and recommendation communication style [32,42].

#### 3.1.2. Building Rapport between Parents and Providers

Having trust is a crucial factor in decreasing vaccine hesitancy. Regardless of race and ethnicity, individuals who reported greater trust in health information provided by HCPs were more likely to vaccinate their children [25]. Studies found that parents and guardians of adolescents consistently reported that doctors are a highly trusted source of information and advice and serve as a key influence in their HPV vaccine decision-making process for their children [25]. Furthermore, studies on vaccination among racial and ethnic minoritized groups found that discussing the vaccine with their HCP was associated with an increased likelihood of vaccination [43,44]. To improve interpersonal connection, the U.S. Centers for Disease Control and Prevention (CDC) recommends that HCPs provide personal examples of supporting the vaccine for their family members and children to make parents more comfortable vaccinating their children [45]. Another best practice is leaving adequate time and opportunities for parents to ask questions and address parental concerns fully. This has been linked to increased parental satisfaction with vaccine communication, reducing vaccination delays and refusals [46].

Often during communication opportunities, parents and/or their children may voice their mistrust of HCPs, the vaccine’s safety and effectiveness, or even their biases about vaccines. To address mistrust, HCPs could question their own implicit and explicit biases and work to build stronger interpersonal trust. As part of patient-centered care, HCPs should express to families that they acknowledge families’ concerns, rather than asserting their competence [47,48]. HCPs can also display their comprehension by verbally affirming their commitment to exploring all possible avenues to address health concerns [47]. By applying the principles of patient-centered communication through active listening while interviewing, patient-centered agenda, and expressing empathetic dialogue, HCPs can create an environment of trust and respect, empowering families to make informed decisions about their health [49]. Even when HCPs disagree with the patient’s perspective, HCPs should show empathy for the patient’s viewpoint and use constructive dialogue to co-create a health solution that includes the patient’s perspective [48]. A trust-centered dynamic strategy is vital to improving patient–provider rapport, consequently improving the likelihood of vaccine uptake.

### 3.2. Content

#### 3.2.1. Persistent Persuasive Language with Minimal Acquiescence

To increase HPV vaccine uptake, simple language with minimal medical jargon is effective [50]. HCPs should convey a clear and unambiguous message supporting the HPV vaccine. A strong recommendation on the need to vaccinate adolescents has been associated with parents perceiving greater urgency to vaccinate their child, greater trust in HCP information, lower vaccine hesitancy, and higher vaccine uptake [51,52]. In contrast, a weak recommendation has been associated with lower vaccine initiation and completion rates among stigmatized racial and ethnic populations [17]. Parents often rely on HCPs to initiate discussions on HPV immunization and tend to comply with their recommendations [41,53]. Receiving a hesitant provider recommendation can be a barrier to vaccine uptake [27]. Research has found that even in parents who have strong convictions to immunize their child for HPV, the lack of HCP endorsement or recommendation to delay vaccination can result in the parent becoming confused, causing them to delay vaccination or even choosing not to vaccinate their child [53,54]. The literature has shown that some parents who intended only to delay vaccination often report ultimately not vaccinating their child due to a lack of provider recommendation [55,56].

Given the importance of effective vaccine recommendation communication, hesitant language that decreases vaccine uptake, including words such as that the vaccine is “elective”, “an option”, or “a potential conversation”, should be minimized by HCPs [54,55]. Instead of hesitant language, HCPs should reframe their communication to a more persuasive language style. Effective vaccine endorsement language is more assertive and includes HCPs stating that: they “strongly recommend” the HPV vaccine, it is a “routine vaccine that their child is due for”, and that the vaccine “was proven safe to prevent cancer” [32,54]. HCPs should continue to use this strong, assertive communication style to promote the vaccine despite parental hesitation. Research shows that, even when parents expressed strong vaccine hesitancy, HCPs who persisted in using assertive vaccine communication had higher vaccine uptake than HCPs who acquiesced [57]. HCPs can use parental hesitancy as a time to engage in patient-centered communication and to tailor the vaccine-related messaging conversation to reduce hesitancy. However, HCPs should still provide respectful communication, even if their patient opts to delay or refuses vaccination, as shaming may cause further resistance and negative patient attitude towards both the vaccine and the HCP [58].

#### 3.2.2. Reframe Conversation Focus from Sex to Cancer

Some parents may be uncomfortable initiating HPV discussions, as they believe the conversation will be around sex [27]. Many parents of stigmatized racial and ethnic minority groups were more supportive of discussing the vaccine when their child’s HCP initiated the conversation and promoted its benefits [41,59]. Lack of perceived HPV risk, susceptibility, and severity may reduce the parents’ perceived benefit of the vaccine, increasing their reluctance to vaccinate their child [60]. When focusing HPV vaccine language on sex, some parents may want to delay vaccination in relation to their child’s young age, despite recognizing that the timing of sexual debut is difficult to predict [55,60]. Prioritizing communication on the benefits of the HPV vaccine as it relates to contracting a sexually transmitted infection (STI) is less appropriate for parents who presume that their child is not sexually active [43,61]. Some parents are also afraid that the HPV vaccine would promote sexual activity for daughters, limiting their willingness to vaccinate their children [40]. It is important to remind parents that research shows no increase in sexual activity following HPV vaccination among adolescents [62].

Shifting the conversation away from sex and to the severity of HPV-related cancers is more effective, as people are more susceptible to messaging about the HPV vaccine as a cancer prevention tool than an STI prevention tool [24,63]. Furthermore, the HPV vaccine conversation should not solely focus on cervical cancer. Some parents are unclear about the need to vaccinate boys, as the initial HPV recommendations focused only on vaccinating girls [64], causing parents to believe that their sons were not at risk for HPV-related cancers [22,41,59]. Hence, it is vital to remind parents that HPV can have adverse health outcomes in males, such as genital warts, and that almost half of HPV-related cancer cases occur in men [3]. Additionally, sexually active men can transmit an HPV infection to their partners. Therefore, emphasizing the personal and altruistic cancer prevention benefits of the HPV vaccine for males and females may boost vaccine uptake.

### 3.3. Modality

#### 3.3.1. Use Multiple Vaccine Reminder Modalities

Research shows that utilizing multiple vaccine reminder sources, such as texting a child’s parent and having reminders in the clinic space, is necessary to encourage vaccine discussion and uptake [65,66,67].

Reminding parents who may have forgotten or were unaware of the HPV vaccination schedule increases vaccine uptake [68]. Short, culturally adapted, informational, and persuasive text message reminders promote vaccine uptake [69,70] and timely vaccine completion among marginalized racial and ethnic groups [71,72]. Within the clinic space, effective communication materials may include showing parents and adolescents interactive videos about HPV to prompt patient–provider vaccine communication [73]. Additionally, it is essential to have physical sources of information, such as brochures with visual and narrative elements, that include HPV infection and HPV vaccine information for different health literacy levels, along with the representation of the intended audience. Bilingual educational materials are vital, as foreign-born parents may find it difficult to obtain comprehensible information on the vaccine in the language and format they need [59,74]. Educational materials can reinforce provider recommendations, particularly when the materials are tailored to the population of interest [75]. Studies have found that individuals preferred HPV visuals with racially diverse representation, and in particular, materials with their community’s image resonated with them more [22,76]. Seeing cultural representation in the reminders may help the parent and child develop an increased awareness of the messages’ relevance. Thus, communication materials should be linguistically, culturally, and visually tailored to the target audience.

Targeted reminders that cue HCPs to recommend the HPV vaccine are also important. HPV vaccine prompts, such as door signs and posters promoting the vaccine, neon vaccine information statement forms, and editing note templates to document refusals and prompt education resources may help remind HCPs to discuss the HPV vaccine with the family [77]. Electronic health record (EHR) systems can be used to prompt vaccine recommendations. EHR-targeted reminders with active choice mechanisms, such as requiring a clinician to actively choose to accept or cancel vaccines, significantly increased vaccination rates [78]. Research has found that EHR prompts also considerably reduced disparities in HPV vaccine uptake between black and white patients, potentially because prior to EHR vaccine prompts, HCPs were less likely to discuss the HPV vaccine with black than white families [66]. Lastly, many HCPs have found it helpful to follow the CDC recommended “same day, same way” approach to vaccination, meaning bundling the HPV vaccine recommendation into the HCP’s discussion of other vaccines that the child needs and vaccinating the child on the same day their parents or guardian approve of the vaccines [45,79].

#### 3.3.2. Work with the Local Community to Culturally Adapt Vaccination Messaging

Social norms are essential to HPV vaccine uptake. Homophily mediates an individual’s vaccine attitude, acceptability, and uptake [80]. Hence, effective HPV vaccine communications need to prioritize community input and foster the co-development of vaccine communication solutions. Local credible messengers may help HCPs improve their approach to discussing the HPV vaccine by ensuring that the messaging and delivery are culturally appropriate, thus increasing the message’s receptivity. There is no one-size-fits-all solution to improving HPV vaccination, as each cultural and social group needs a unique set of tailored intervention strategies. Even people who identify as the same race and ethnicity can have heterogeneous backgrounds and norms, which can influence vaccine acceptance [40]. Consequently, in health program communications and materials, it is crucial to work with the local community and understand the specific barriers to vaccine uptake within that community, using community-based participatory principles [74,81,82]. To improve communications, HCP cultural competence conversations and training should include local community leaders, faith-based leaders, and health educators [83]. Additionally, diverse representation among HCPs is vital, as racial and ethnic congruence is associated with more trusting HCP–patient relations, since it encourages a positive homophily effect [17,25].

For vaccine-hesitant parents, providing facts on HPV and the vaccine is not enough. Research showed that vaccine-hesitant parents were more likely to vaccinate when the HCP created a more personalized vaccine recommendation with a collaborative conversational style [33]. However, patients from stigmatized racial and ethnic groups often experience less collaborative communication during patient–provider communication, potentially due to perceived HCP difficulties in cross-cultural communication [84]. To address the communication barrier, HCPs should build their skills in collaborative communication, including engaging in cross-cultural collaborative conversations. Understanding the community through feedback from community health workers and parents is vital to forming credible and suitable personalized recommendations for community members [33,85]. Developing partnerships with local religious groups and community-based organizations to deliver HPV vaccine information can build a foundation of patient–provider trustworthiness and address structural medical mistrust over time [22,25]. Greater community dialogue may lead to changes in social norms and encourage family and friends to openly promote the vaccine [60]. Linguistically and culturally tailored health education community forums that include community leaders show community support for the vaccine, which is vital to enhancing trust, vaccine acceptance, and vaccine uptake [86,87]. Greater work on creating constructive dialogues between the community and HCPs is crucial to helping HCPs better tailor the vaccine information to the community’s needs and concerns.

## 4. Discussion

HCPs need to recognize the importance of their recommendation in encouraging HPV vaccine uptake. Based on the review, HCPs can utilize a set of language strategies to improve HPV vaccine communication and uptake. These key vaccine communication language and strategies HCPs could use and avoid using when recommending the HPV vaccine to adolescents and their families are outlined in Table 1.

Overall, while providing knowledgeable and concise explanations about the HPV virus and vaccine, HCPs should maintain a persistent and persuasive vaccine recommendation language. Additionally, HCPs should provide patient-centered communications through active listening and communicate with culturally and religiously adapted vaccine language co-developed with the local community. HCPs should also utilize multiple culturally adapted vaccine reminders in various modalities to prompt patient–provider HPV discussion. There is also a set of language that HCPs should minimize when discussing the vaccine, such as shaming patients for their vaccine refusal and utilizing hesitant vaccine recommendation language. Framing vaccine uptake from one’s perspective without adapting the content to the patient’s culture, religion, health literacy level, and personal views may also decrease HPV vaccine uptake. Additionally, HCPs should not avoid patient questions about vaccine risks, ignore their concerns about medical mistrust, or shame patients for delaying or refusing the vaccine.

In sum, to promote equitable HPV vaccine uptake, health stakeholders should create HCP educational workshops that prioritize HCPs’ skills regarding communicating HPV vaccine information and recommendation in a culturally competent and patient-centered manner.

### 4.1. Limitations

Many of the studies reviewed had methodological limitations. For example, several studies used data collection methods with a high risk of self-report bias. The classifications of race and ethnicity vary across studies; hence, it may be difficult to compare culturally-specific barriers and successes of HPV vaccination across studies. This article focuses on the interpersonal level and the role of patient–provider communication; however, the authors acknowledge the importance of social norms related to child vaccination, which impact vaccination uptake above and beyond the factors examined. The authors also recognize that successful HPV vaccine programs and the reduction in cervical cancer mortality require an integrative effort beyond effective patient–provider communication, including increased access to care, affordability of care, and system-based changes. Additionally, stigmatized racial and ethnic groups are heterogenous, with large differences within each group. Nevertheless, the authors have found general themes and considerations to adapting HPV vaccination that may be similar across different groups. The authors hope this article is an initial step to highlighting the critical need for greater awareness, research, and implementation of equitable HPV vaccine uptake.

### 4.2. Future Directions

There are gaps in the research-based evidence on how to adapt vaccine communications culturally to better reach adolescents of color and further test potential effect differences in messaging strategies. The recommendations include testing various motivational and persistent communication languages mentioned above and summarized in Table 1 to understand potential variations in efficacy, particularly among different cultural groups. As time constraints are a common barrier in patient–provider vaccine communication, future research should test the effectiveness of brief strategies to efficiently increase vaccine uptake in different scenarios, including healthcare, community, and school settings. Studies should also test the effectiveness of various methods of clinician and parent HPV vaccine reminders, such as the effects of various media-based interventions [88].

There are many federal campaigns and programs that have promoted vaccine uptake, and these activities have contributed to changing social norms related to child vaccination [89]. The health communications recommendations in this article can be utilized beyond patient–provider communication and can be incorporated to address the current gaps in HPV vaccine campaigns. The communications concepts and strategies highlighted above should be further tested to improve equitable and culturally competent health communication in all settings.

## 5. Conclusions

Given the disproportionate burden of HPV-related cancers among stigmatized racial and ethnic groups, culturally sensitive vaccine recommendations that consider source, content, and modality communication components are vital for HCPs when establishing a rapport with their patients. HCPs need to communicate in a way that is linguistically and culturally appropriate, as well as in an environment where adolescents and their parents can feel comfortable voicing their concerns. It is imperative that public health stakeholders, particularly HCPs, recognize the gaps in knowledge in providing HPV vaccine recommendations and care to stigmatized racial and ethnic groups. HCPs should utilize their position as a highly trusted source of health information to recommend the HPV vaccine in a systematic manner to all relevant patients and strive to administer the vaccine within the same visit. Strategies to increase vaccine uptake include strongly and persistently recommending the vaccine and emphasizing the vaccine’s cancer prevention benefits for both males and females. Additionally, having repeated vaccine recommendation prompts in multiple modalities and co-creating vaccine messaging with local communities may improve HPV vaccine communication for marginalized racial and ethnic groups. The need to provide culturally competent healthcare communication is dire. Failure to provide equitable patient–provider vaccine communication may exacerbate racial and ethnic disparities in cervical cancer and other HPV-related diseases.

## Figures and Tables

**Table 1:** HPV Vaccine Communication Language to Use and Avoid by Health Care Providers.

HPV Vaccine Communication Language to Use and Avoid by Health Care Providers	
**Language to use:**	- Provide knowledgeable and concise explanations about the HPV virus, as well as the safety and recommendation guidelines for the HPV vaccine. - Confirm the patient has received the complete dosing of the vaccine for their appropriate vaccine initiation age and reiterate the importance of complete dosing to optimize vaccine uptake and reduce risk of disease. - Provide patient-centered communications through active listening, a patient-centered agenda, and expressing empathy during the patient–provider discussion. - Utilize concrete personal examples of the vaccine’s effectiveness to build patient–provider rapport and trust. - Maintain persistent and persuasive vaccine recommendation language with minimal acquiescence. - Focus vaccine language on cancer-related outcomes, instead of solely on sex, to enhance risk perception. - Repeat culturally adapted vaccine reminders in multiple modalities to prompt patient–provider HPV discussion. - Communicate with culturally and religiously adapted vaccine language co-developed with the local community. - Repeat vaccine recommendations during later meetings, despite the patient expressing a decision to delay vaccine uptake or refusing the vaccine. - Participate in educational workshops that prioritize providers’ skills on communicating about the HPV virus and the HPV vaccine in a culturally competent and patient-centered manner.
**Language to avoid:**	- Limited explanation of the HPV vaccine because of perceived potential refusal and/or lack of provider vaccine communication self-efficacy - Shaming patients for vaccine refusal - Utilizing hesitant vaccine recommendation language - Framing vaccine uptake from one’s perspective without adapting content to the patient’s culture, religion, and personal views - Avoiding patient questions about vaccine risks and lack of easing medical mistrust - Limited vaccine educational materials in various appropriate languages, formats, visual imagery, and health literacy levels

## Data Availability

Not applicable.
